# The complete chloroplast genome sequence of *Lemna turionifera* (Araceae)

**DOI:** 10.1080/23802359.2024.2384577

**Published:** 2024-07-31

**Authors:** Jiexin Lin, Zhongyuan Lin, Yanqiong Chen, Huibin Xu

**Affiliations:** Fuzhou Institute of Oceanography, Minjiang University, Fuzhou, China

**Keywords:** Duckweed, *Lemna turionifera*, chloroplast genome, phylogeny

## Abstract

*Lemna turionifera* is native to North America and northern Asia, with significant potential for industrial wastewater remediation. The complete nucleotide sequence of the *L. turionifera* chloroplast genome (cpDNA) was determined. The cpDNA is a circular molecule of 166,606 bp and containing a pair of inverted repeats (IRs) measuting 31,663 bp each. These IRs are flanked by a small single-copy region of 13,542 bp and a large single-copy region of 89,738 bp. The chloroplast genome of *L. turionifera* consisted of 112 unique genes, including 78 protein-encoding genes, 30 tRNA genes, and four rRNA genes. The phylogenetic analysis utilizing cpDNA provided a well-supported resolution of the relationships among subfamilies within the Araceae family. Our findings indicated a close relationship between *L. turionifera* and a clade consisting of *L. minor*, *L. japonica*, and *L. gibba*. The availability of the complete chloroplast genome sequence of *L. turionifera* presents valuable data for future phylogenetic investigations within the Lemnaceae family.

## Introduction

Duckweed, belonging to the subfamily Lemnaceae (Araceae), is a small aquatic flowering monocotyledonous plant with five genera (*Spirodela*, *Landoltia*, *Lemna*, *Wolffia* and *Wolffiella*) comprising 37 species (Landolt 1986; Les et al. 2002). It holds significant potential for biotech applications, particularly in wastewater remediation. Studying the duckweed genome is crucial for a comprehensive understanding of its genomic composition and regulatory elements. Morphological characters have traditionally been used for taxonomic and phylogenetic studies in Lemnoideae, but their small size and morphological degeneration make phylogenetic relationships within different genera challenging to resolve. Chloroplast genomes (cpDNAs), with their rich genetic information, have been employed to clarify the phylogeny within Lemnoideae. While chloroplast genome sequences have been reported in all Lemnoideae genera, species coverage varies (Mardanov et al. 2008; Wang & Messing 2011; Ding et al. 2017; Zhang et al. 2020). For instance, among the 13 species in the *Lemna* genus, only three species (*Lemna minor* L. 1753, *L. japonica* L. 1753, and *L. gibba* L. 1753) have been studied (Mardanov et al. 2008; Ding et al. 2017; Yang et al. 2021). *Lemna turionifera* Landolt 1975, native to North America and northern Asia, exhibits rapid nitrogen and phosphorus removal in municipal wastewater, making it an excellent candidate for future wastewater treatment applications when integrated into the purification systems (Zhou et al. 2018). In this research, we present the first complete chloroplast genome sequence of *L. turionifera* and construct a phylogenetic tree, providing a scientific basis for understanding its taxonomic status.

## Materials and methods

**Figure 1.**
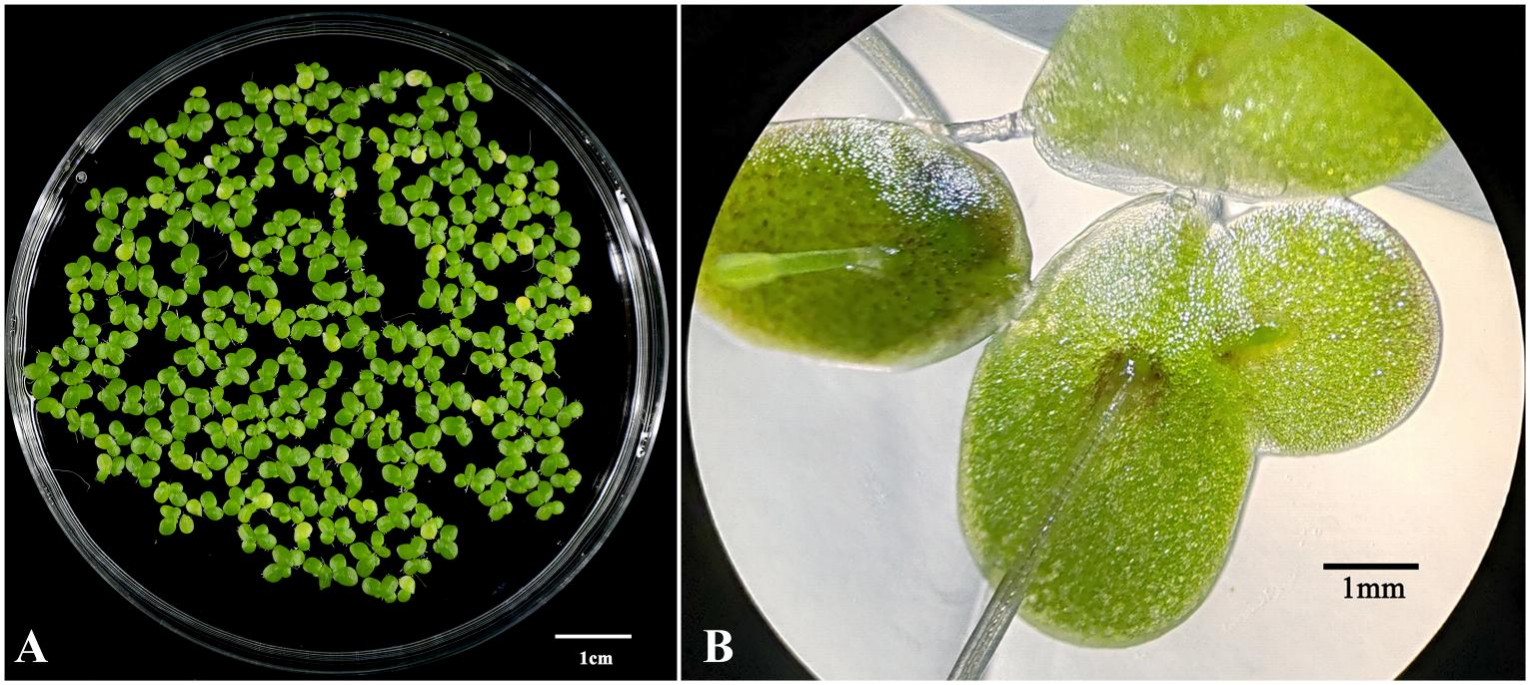
Species reference image of *lemna turionifera* (image was taken by Huibin Xu in Qingdao, Shandong, China, 120°36’N, 36°57’E). (A) frontal view (the thallus is obovate, egg-shaped, but with the widest point above the Middle of the leaf blade), (B) dorsal view (with red to purple coloration on lower surface).

Total DNA was isolated from fresh leaves of *L. turionifera* collected from Qingdao, Shandong Province, China (120°36’N, 36°57’E) (Figure 1). The DNA was deposited in the Marine and Agricultural Biotechnology Laboratory, Minjiang University, under the DNA number LT6002 (Fuzhou, China; Huibin Xu, xuhuibin@mju.edu.cn). Experimental research and field studies on plants comply with relevant institutional, national, and international guidelines and legislation.

Total DNA was sent to Novogene Co., LTD (Beijing, China) for library preparation and paired-end (PE) sequenced by the Illumina NovaSeq 6000. The raw PE reads were assembled into contigs using the GetOrganelle pipeline (Jin et al. 2020). Contigs were then connected into the plastome using Bandage (Wick et al. 2015). The annotation of the chloroplast genome was performed through the online program CPGAVAS2 (Shi et al. 2019) and GeSeq (Tillich et al. 2017). The start and stop codons of protein-coding genes were manually adjusted by comparison with other plastid genomes of Araceae from Genbank (e.g. *L. minor* DQ400350) using Geneious prime v2022.1.1. The physical map of the plastome was generated by the online service Organellar Genome DRAW tool (OGDRAW) (Greiner et al. 2019) with default parameters and edited manually.

To explore the phylogenetic relationship of *L. turionifera* within the family Araceae, chloroplast genome sequences of 32 species from the family Araceae were randomly downloaded, and three species from the genera *Tofieldia* were set as outgroups from GenBank. The sequences were aligned using MAFFT v7.310 (Katoh and Standley 2013). RAxML-HPC2 on XSEDE version 8.2.10 (Stamatakis 2014) was used to construct a maximum likelihood tree. The junction regions between the IR, SSC, and LSC of *L. mino*r and *L. turionifera* were compared using the online program IRscope (https://irscope.shinyapps.io/irapp/) (Amiryousefi et al. 2018).

## Results

**Figure 2.**
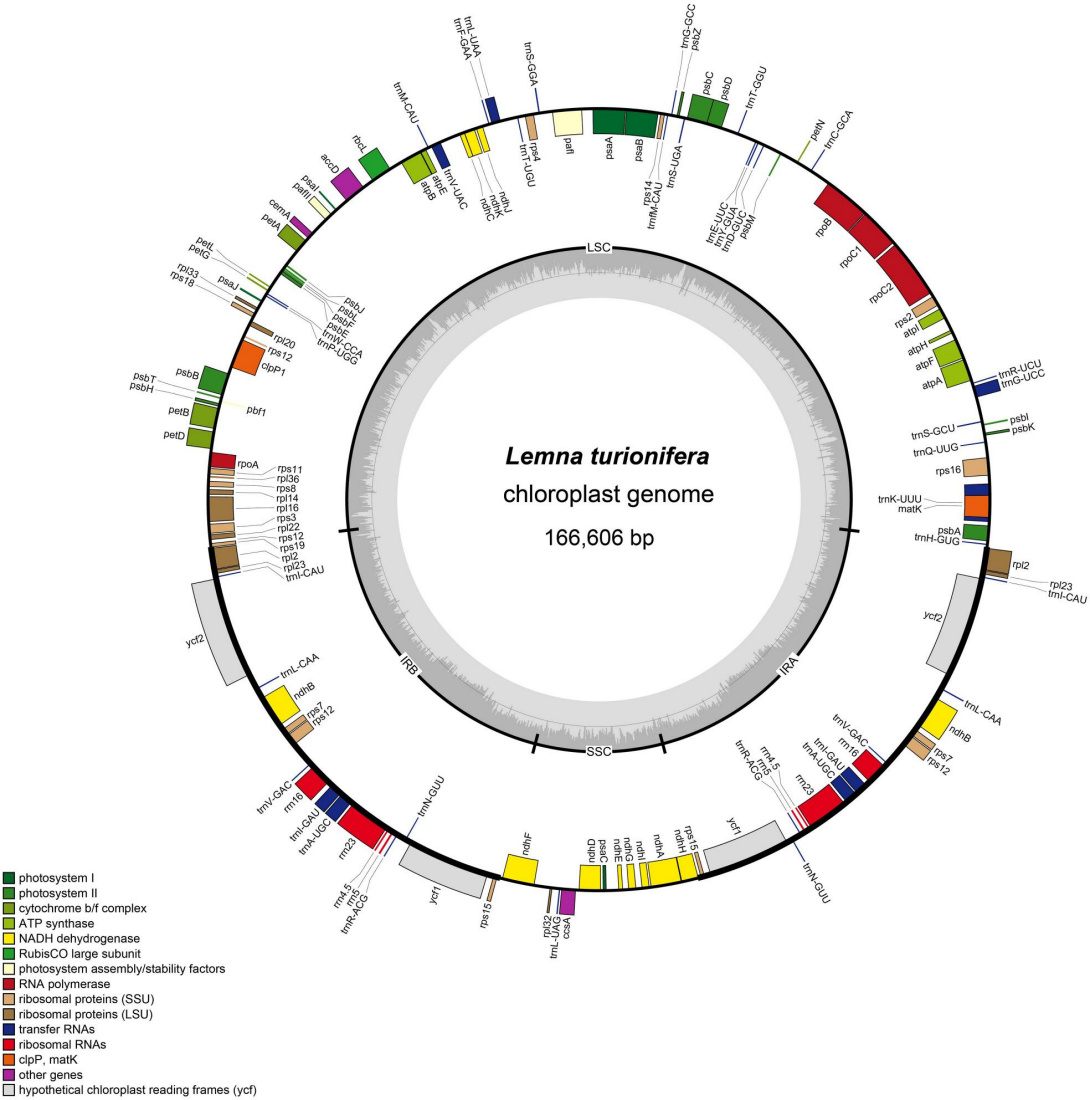
Chloroplast gene map of *L. turionifera*. Genes on the outside and inside of the circle are transcribed in the clockwise and counterclockwise directions, respectively. The dark and light gray bars in the inner circle denote G + C and A+ T contents, respectively.

The coverage of the genome from paired-end reads was 100 %, with an average depth of 1379.78. Despite the presence of certain loci with low coverage, the assembly successfully generates a complete circular configuration of the chloroplast genome (Figure S1). The complete chloroplast genome of *L. turionifera* is 166,606 bp in size, with 35.8 % of the overall GC content. It contains a pair of inverted repeats (IRs) with 31,663 bp for each, which are separated by a large single-copy region (LSC) of 89,738 bp and a small single-copy region (SSC) of 13,542 bp (Figure 2). The chloroplast genome contains 112 unique genes, including four ribosomal RNA (rRNA) genes, 30 transfer RNA (tRNA) genes, and 78 protein-coding genes. We identified 13 cis-splicing genes and the trans-splicing gene *rps12*, and their structures are shown in Figure S2. Most genes are single copies in the SSC or LSC, whereas four rRNA genes, seven tRNA genes, and eight protein-coding genes have two copies.

**Figure 3.**
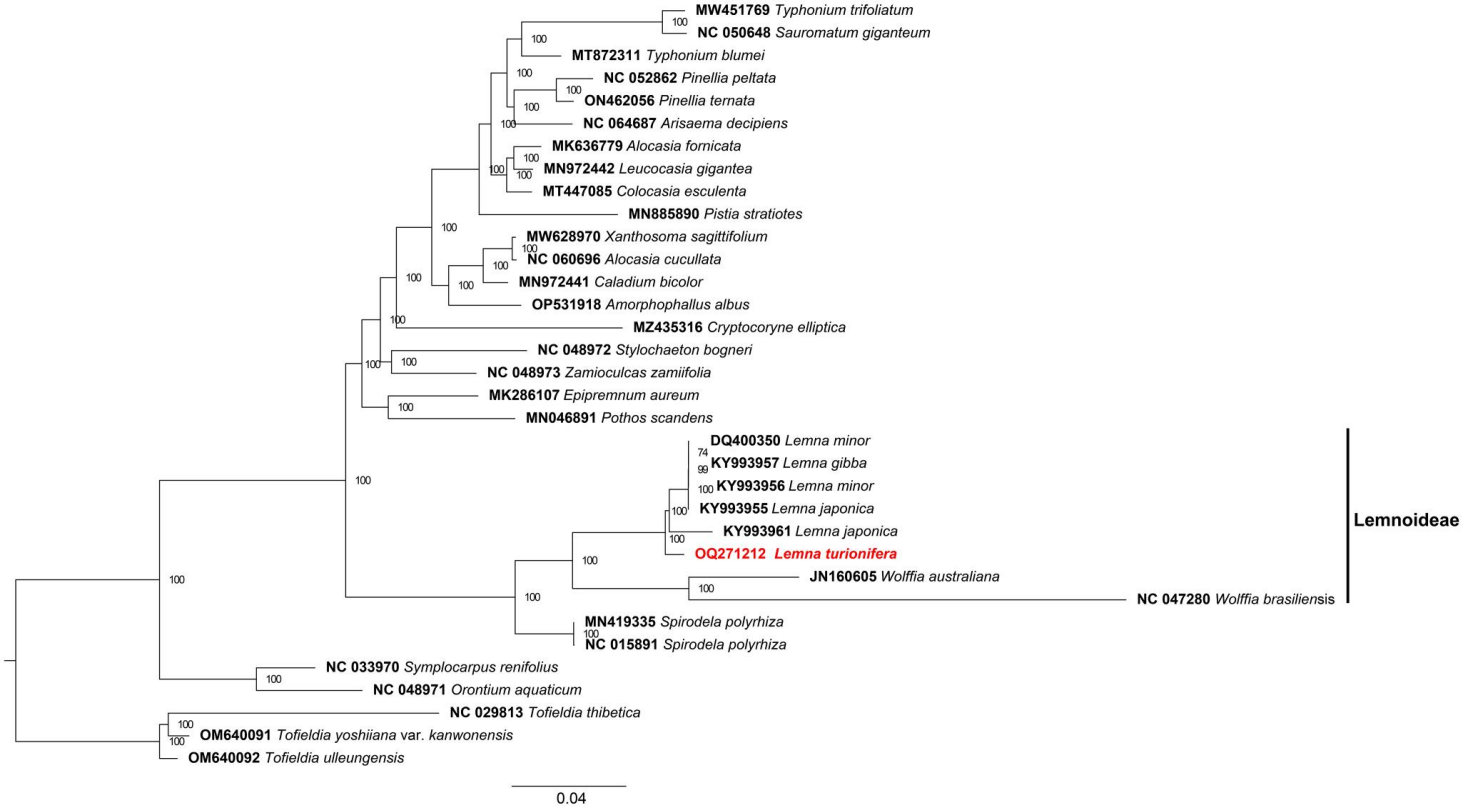
Maximum likelihood tree based on the complete chloroplast genome sequences of 27 species from the Araceae, with three Tofieldia species as outgroup. Shown next to the nodes are bootstrap support values based on 1000 replicates. The scale bar means the expected number of nucleotide substitutions per site. The following sequences were used: *Lemna japonica* KY993961 (Ding et al. 2017), *Lemna gibba* KY993957 (Ding et al. 2017), *Lemna minor* KY993956 strain 9532 (Ding et al. 2017), *Lemna japonica* KY993955 (Ding et al. 2017), *Lemna minor* DQ400350 (Mardanov et al. 2008), *Wolffia australiana* JN160605 (Wang and Messing 2011), *Epipremnum aureum* MK286107 (Guan et al. 2019), *Alocasia fornicata* MK636779 (Abdullah et al. 2021), *Pothos scandens* MN046891 (Abdullah et al. 2020a), *Spirodela polyrhiza* MN419335 (Zhang et al. 2020), *Pistia stratiotes* MN885890 (Quan and Chen 2020), *Caladium bicolor* MN972441 (Zhang et al. 2018), *Leucocasia gigantea* MN972442 (Zhang et al. 2018), *Colocasia esculenta* MT447085 (unpublished), *Typhonium blumei* MT872311, *Typhonium trifoliatum* MW451769(unpublished), *Xanthosoma sagittifolium* MW628970 (Low et al. 2021), *Cryptocoryne elliptica* MZ435316 (Talkah et al. 2022), *Spirodela polyrhiza* NC015891 (Wang and Messing 2011), *Tofieldia thibetica* NC029813 (Luo et al. 2016), *Symplocarpus renifolius* NC033970 (Choi et al. 2017), *Wolffia brasiliensis* NC047280 (Park et al. 2020), *Orontium aquaticum* NC048971 (Abdullah et al. 2020b), *Stylochaeton bogneri* NC048972 (Abdullah et al. 2020b), *Zamioculcas zamiifolia* NC048973 (Abdullah et al. 2020b), *Sauromatum giganteum* NC050648 (Kim et al. 2020), *Pinellia peltata* NC052862 (Luo et al. 2016), *Alocasia cucullata* NC060696 (Low et al. 2021), *Arisaema decipiens* NC064687 (unpublished), *Tofieldia yoshiiana* var. *kanwonensis* OM640091 (unpublished), *Tofieldia ulleungensis* OM640092 (unpublished), *Pinellia ternata* ON462056 (unpublished), *Amorphophallus konjac* OP531918 (unpublished).

The best-fit model of nucleotide substitution according to BIC was GTR + I + G. The phylogenetic tree based on these chloroplast genomes showed the relationship between different subfamilies in Araceae was resolved with mostly high support, *L. turionifera* is closely related to the clade formed by *L. minor, L. japonica* and *L. gibba* (Figure 3). Between the *L. minor* and *L. turionifera*, the gene positions of four borders (LSC/IRb, IRb/SSC, SSC/IRa, and IRa/LSC) had different types. In the *L. minor*, the *rpl2* overlapped the LSC/IRb, while in the *L. turionifera* was *rps19* (Figure S3).

## Discussion

Species of duckweed hold great potential for biotech applications, including efficient wastewater remediation. However, inconsistencies in previous studies regarding the phylogenetic relationships and taxonomic systems of wild duckweed species can impact their applications. Identifying *Lemna* species based on morphology alone is challenging due to variations in frond and root shapes (Dudley 1987). Moreover, morphological traits are easily influenced by the environment, leading to convergence and parallel evolution (Xing et al. 2013). Genomic information availability enhances research possibilities for diverse duckweed species. DNA studies provide reliable molecular evidence for understanding the phylogenetic evolution of species and identifying similar species within a genus.

Studying chloroplast genomes is important for understanding chloroplast DNA structure, origin, plant molecular markers, and species relationships (Tang et al. 2011). Sequencing and analyzing the chloroplast genomes of duckweed species contribute to expanding the available chloroplast genome sequences, enabling species identification, studying phylogenetic relationships, developing improved varieties, and sustainably utilizing plant resources. Therefore, future research should prioritize the systematic collection of *Lemna* specimens and the execution of comprehensive investigations into its nuclear and organelle phylogenies. These endeavors are essential for elucidating the precise phylogenetic relationship of *Lemna* and advancing our understanding in this field.

## Conclusion

The chloroplast genome of *L. turionifera* was assembled using short-read data in this study. It showed a typical tetrameric structure, similar to the chloroplast genomes of other plants in the family Aracea. The phylogenetic tree demonstrated a clear relationship between *L. japonica, L. minor,* and *L. gibba.* Thus, our findings not only offer vital genetic resources of *Lemna* but also provide insights into the evolution of the species.

## Supplementary Material

Supplemental Material

Supplemental Material

Supplemental Material

## Data Availability

The genome sequence data that support the findings of this study are openly available in GenBank of NCBI at (https://www.ncbi.nlm.nih.gov/) under the accession no. OQ271212 (https://www.ncbi.nlm.nih.gov/nuccore/OQ271212.1/). The associated BioProject, Bio-Sample, and SRA numbers are PRJNA947687, SAMN33865469, SRR23943402, respectively.
