# Supplementary figures and images for: The complete chloroplast genome sequence of Lemna turionifera (Araceae)
Source: Mitochondrial DNA B Resour. 2024 Jul 31;9(8):971–5. doi: 10.1080/23802359.2024.2384577 (PMC11293259; doi:10.1080/23802359.2024.2384577)

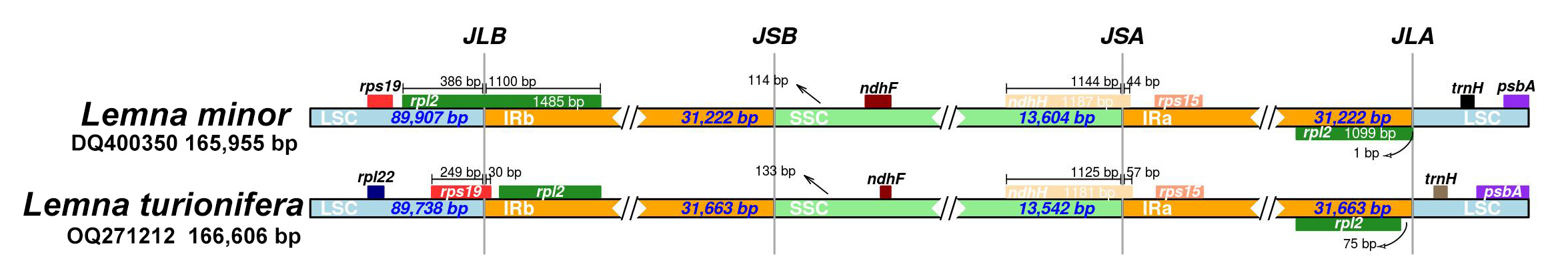

Supplement: Supplemental Material [file TMDN_A_2384577_SM1298.jpg]

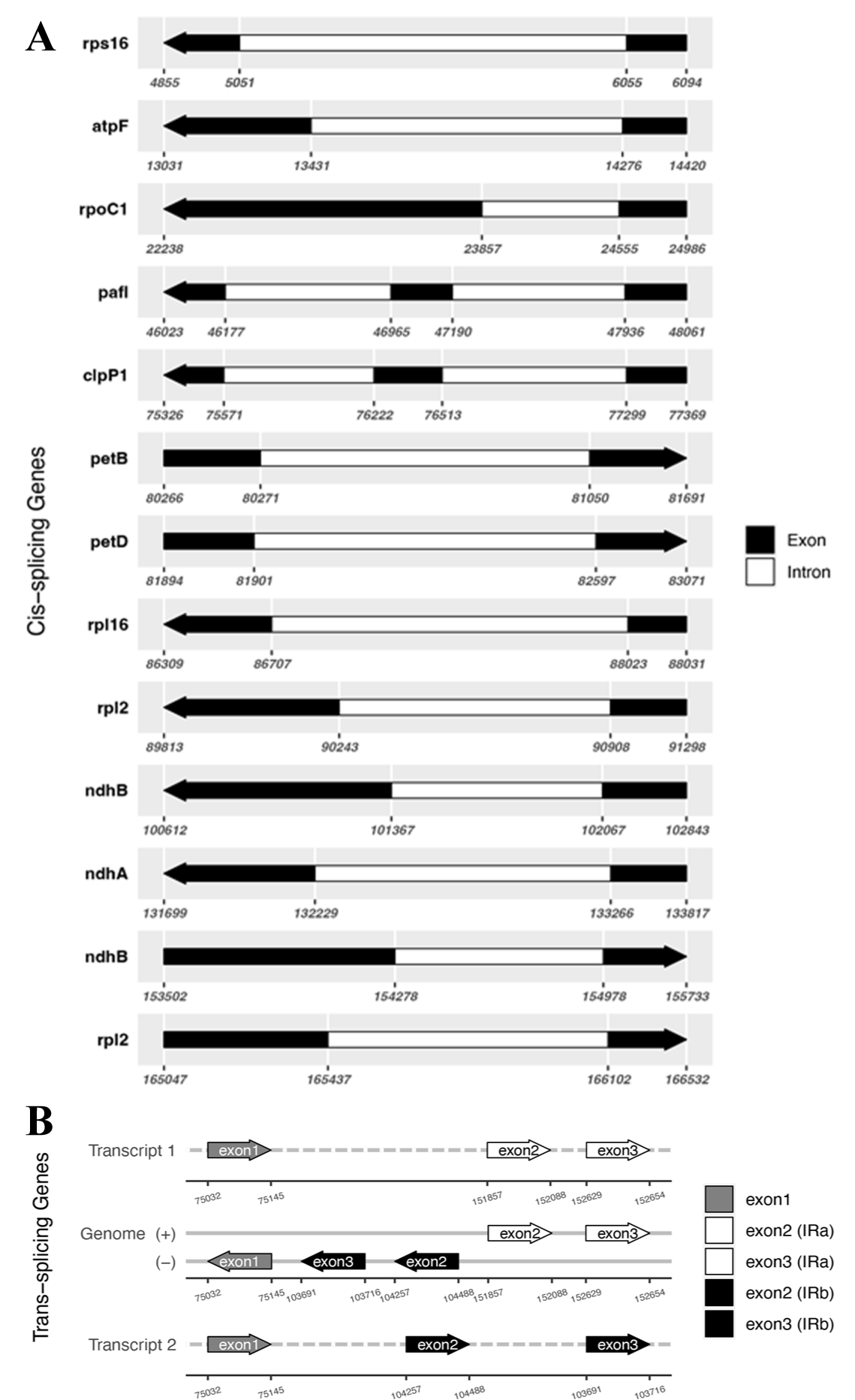

Supplement: Supplemental Material [file TMDN_A_2384577_SM1297.png]

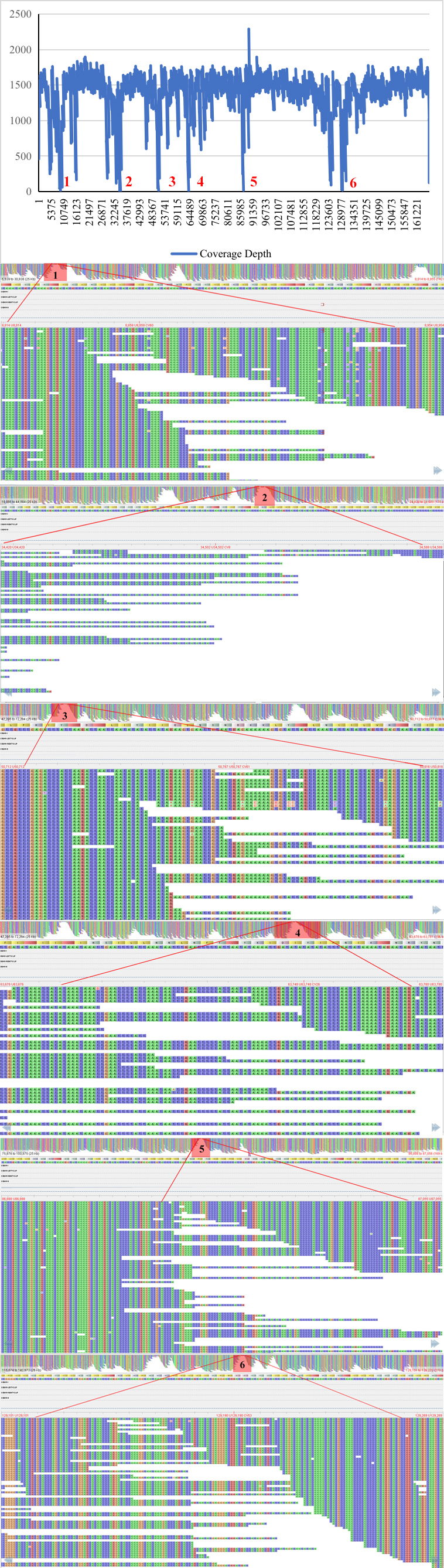

Supplement: Supplemental Material [file TMDN_A_2384577_SM1296.jpg]
